# The Peptidoglycan-associated lipoprotein Pal contributes to the virulence of *Burkholderia mallei* and provides protection against lethal aerosol challenge

**DOI:** 10.1080/21505594.2020.1804275

**Published:** 2020-08-15

**Authors:** Jeremy S. Dyke, Maria Cristina Huertas-Diaz, Frank Michel, Nathan E. Holladay, Robert J. Hogan, Biao He, Eric R. Lafontaine

**Affiliations:** a Department of Infectious Diseases, University of Georgia College of Veterinary Medicine, Athens, GA, USA; b Department of Veterinary Biosciences and Diagnostic Imaging, University of Georgia College of Veterinary Medicine, Athens, GA, USA

**Keywords:** *Burkholderia mallei*, glanders, virulence, mouse aerosol infection, vaccine, protective antigen

## Abstract

Burkholderia mallei

is a highly pathogenic bacterium that causes the fatal zoonosis glanders. The organism specifies multiple membrane proteins, which represent prime targets for the development of countermeasures given their location at the host-pathogen interface. We investigated one of these proteins, Pal, and discovered that it is involved in the ability of *B. mallei* to resist complement-mediated killing and replicate inside host cells *in vitro*, is expressed *in vivo* and induces antibodies during the course of infection, and contributes to virulence in a mouse model of aerosol infection. A mutant in the *pal* gene of the *B. mallei* wild-type strain ATCC 23344 was found to be especially attenuated, as BALB/c mice challenged with the equivalent of 5,350 LD_50_ completely cleared infection. Based on these findings, we tested the hypothesis that a vaccine containing the Pal protein elicits protective immunity against aerosol challenge. To achieve this, the *pal* gene was cloned in the vaccine vector Parainfluenza Virus 5 (PIV5) and mice immunized with the virus were infected with a lethal dose of *B. mallei*. These experiments revealed that a single dose of PIV5 expressing Pal provided 80% survival over a period of 40 days post-challenge. In contrast, only 10% of mice vaccinated with a PIV5 control virus construct survived infection. Taken together, our data establish that the Peptidoglycan-associated lipoprotein Pal is a critical virulence determinant of *B. mallei* and effective target for developing a glanders vaccine.

## Introduction

*Burkholderia mallei* is a host-adapted Gram-negative bacterium and causes the incapacitating and highly fatal zoonotic disease glanders, which affects primarily horses, donkeys, and mules. The organism is extremely contagious through contact with diseased equids and humans are susceptible to infection. Glanders is endemic in Asia, South America, Africa, and the Middle East and the disease is closely monitored by the World Organization of Animal Health as it is considered a biosecurity and biosafety threat [1–3].

Comparative analyses indicate that *B. mallei* evolved from the bacterium *Burkholderia pseudomallei* through genomic reduction. The latter is commonly found in water and wet soils of countries bordering the equator and causes the global emerging tropical disease melioidosis [4–6]. The genes retained by *B. mallei* have a high level of sequence identity with their *B. pseudomallei* orthologs and the two species share many virulence determinants [7–10]. The clinical and pathological manifestations of disease caused by the organisms are also very similar. In humans, infection typically occurs through punctured skin and the respiratory route, and the most common manifestations are life-threatening pneumonia and bacteremia [3,4,11,12]. Pathogenesis is complex and entails the synchronized expression of genes supporting intracellular and extracellular replication of bacteria, colonization of deep tissues and organ systems, and development of hallmark chronic lesions that are difficult to treat and eliminate [13–16].

Glanders and melioidosis lack reliable modern diagnostic tools and require extended therapy with low success rate due in part to the intrinsic resistance of *B. mallei* and *B. pseudomallei* to antibiotics [17,18]. No vaccine exists to protect animals or humans and there is concern that these highly pathogenic bacteria could be used as bioweapons, hence their classification as Tier 1 Select Agents by U.S federal agencies. Fortunately, the genetic, virulence, and disease similarities between *B. mallei* and *B. pseudomallei* suggest the feasibility of devising medical countermeasures that protect against both organisms. This premise is supported by studies showing that antibodies against *in vivo* expressed *Burkholderia* antigens [19], vaccination with *Burkholderia* live attenuated strains [19,20] and outer membrane vesicles [21,22], and polysaccharide-based vaccines [23,24] provide cross-species protection in animal models of melioidosis and glanders.

Immunoreactive proteins expressed by *B. mallei* and *B. pseudomallei* during human infections have been identified using immunoproteomic approaches and represent high value targets for developing countermeasures [25–28]. One particular *B. pseudomallei* antigen, designated BPSL2765 (locus tag in the genome of strain K96243), was found to induce immune responses linked to lower incidence of chronic and recurrent melioidosis [26]. Immunization with subunit vaccines containing BPSL2765 was also shown to provide partial protection against intraperitoneal challenge in a mouse model of melioidosis [29,30].

Comparative sequence analyses and determination of the crystal structure of BPSL2765 indicated that the protein is an ortholog of the well-characterized Peptidoglycan-associated lipoprotein (Pal) of Gram-negative bacteria [31]. Pal is a component of the Tol-Pal system, which comprises the five core proteins TolQ, TolR, TolA, TolB, and Pal. The Tol-Pal proteins form a complex that spans the inner and outer membranes and is required for maintaining cell wall integrity and can be exploited for the entry of macromolecules such as bacteriocins and bacteriophages [32,33]. The first amino acid of the mature Pal protein is a lipidated cysteine and the hydrophobic N-terminus anchors Pal in the outer membrane while the C-terminal region interacts with the meso-diaminopimelate residue of peptidoglycan in the periplasm. Pal also interacts with the TolB and TolA proteins, which are located in the periplasm and the inner membrane, respectively. The other components of the Tol-Pal system, TolQ and TolR, are located in the inner membrane and interact with TolA. Together, the Tol-Pal proteins form a cross-bridge between the outer membrane, peptidoglycan cell wall, and inner membrane [32,33].

Despite in-depth characterization in many organisms, the biological role of Pal in *B. mallei* and *B. pseudomallei* was not investigated previously and its value as a vaccine target against aerosol exposure has not been determined. The goals of this study were to engineer a *B. mallei pal* mutant strain, investigate the phenotypic traits of the mutant, and develop a delivery system to test the vaccinogenic potential of the antigen in a mouse model of infection.

## Materials and methods

### In silico *analyses*

The ExPASy Bioinformatics Resource Portal (https://www.expasy.org) was used to analyze nucleotide and protein sequences. LipoP 1.0 (http://www.cbs.dtu.dk/services/LipoP/) and SignalP 5.0 (http://www.cbs.dtu.dk/services/SignalP/) were used to identify signal sequence cleavage sites within proteins. Conserved domains were identified with the NCBI Conserved Domain Database (CDD; https://www.ncbi.nlm.nih.gov/cdd/). NCBI BLAST (https://blast.ncbi.nlm.nih.gov/Blast.cgi) was used to search bacterial genomes and identify *pal* orthologs.

### Plasmids, bacterial strains, and growth conditions

Table 1 lists the strains and plasmids used in this study. *Burkholderia mallei* strains were routinely propagated in *Brucella* medium (BD) containing glycerol (5% vol/vol) at a temperature of 37°C. The medium was supplemented with zeocin (7.5 ug/mL), kanamycin (5 ug/mL), and/or Polymyxin B (5 ug/mL) when appropriate. For infection experiments (cell culture, mouse challenge), *B. mallei* was grown on agar plates for 2 days and bacteria were suspended to a concentration of 1 × 10^9^ CFU per mL in phosphate-buffered saline (PBS). The suspensions were then serially diluted and used to infect cell cultures and mice. For all experiments, 100-uL portions of the bacterial suspensions (and dilutions) were spread onto agar plates to determine the number of CFU in the inoculum. *Escherichia coli* strains were cultured in low-salt Luria-Bertani (LSLB) medium at 37°C. The medium was supplemented with chloramphenicol (15 ug/mL), kanamycin (50 ug/mL), zeocin (50 ug/mL), and/or tetracycline (15 ug/mL) when appropriate.

**Table 1.** Strains and plasmids.

Strain or plasmid	Description	Source or reference
*B. mallei*		
ATCC 23344	Wild-type strain; polymyxin B resistant, kanamycin and zeocin sensitive	[10]
*pal* KO	Isogenic *pal* mutant strain of ATCC 23344; resistant to polymyxin B and zeocin, sensitive to kanamycin	This study
*E. coli*		
EPI300	Strain used for general recombinant DNA manipulations; sensitive to kanamycin, zeocin, tetracycline, and chloramphenicol	epicenter/Lucigen
EC100D *pir^+^*	Strain used for recombinant DNA manipulations of plasmid pKAS46 and derivatives; sensitive to kanamycin and zeocin	epicenter/Lucigen
S17	Strain used for conjugational transfer of plasmids to *B. mallei*; sensitive to kanamycin, zeocin, and polymyxin B	[34]
TUNER	Protein expression strain used to purify His-tagged Pal_Bm_ and LolC_Bm_ proteins; chloramphenicol sensitive	Millipore Sigma
Plasmids		
pBHR1	Cloning vector; confers resistance to chloramphenicol and kanamycin	MoBiTec
pBHR1∆ Dra	pBHR1 containing a 339 nt deletion in the chloramphenicol resistance marker; confers resistance only to kanamycin	[43]
pPal_Bm_	pBHR1 in which a 812 nt DNA fragment containing the *pal* gene of *B. mallei* ATCC 23344 was inserted; confers resistance only to kanamycin	This study
pEM7/ZEO	Source of the zeocin resistance cassette; confers resistance to zeocin	ThermoFisher Scientific
pACYC184	Cloning vector; confers resistance to chloramphenicol and tetracycline	New England Biolabs Inc.
pACYCPal_Bm_	pACYC184 in which a 1,332 nt DNA fragment containing the *pal* gene of *B. mallei* ATCC 23344 was inserted; confers resistance to tetracycline	This study
pACYCPal_Bm_ZEO	pACYCPal_Bm_ in which a zeocin resistance cassette was inserted near the middle of the *pal* gene; confers resistance to tetracycline and zeocin	This study
pKAS46	Gene replacement vector; confers resistance to kanamycin	[35]
pKASPal_Bm_ZEO	pKAS46 containing the *pal* gene interrupted with a zeocin resistance cassette from pACYCPal_Bm_ZEO; confers resistance to kanamycin and zeocin	This study
pETcoco-1	His-tagged protein expression vector; confers resistance to chloramphenicol	Millipore Sigma
pHisPal_Bm_	pETcoco-1 in which a gene fragment encoding amino acids 22 to 170 of *B. mallei* ATCC 23344 Pal was inserted; confers resistance to chloramphenicol	This study
pHisLolC_Bm_	pETcoco-1 in which a gene fragment encoding amino acids 47 to 243 of *B. mallei* ATCC 23344 LolC was inserted; confers resistance to chloramphenicol	This study
pMHD11	pZL185 (PIV5 genome) in which a gene fragment encoding amino acids 22 to 170 of *B. mallei* ATCC 23344 Pal was inserted; confers resistance to chloramphenicol	This study
Viruses		
PIV5-Pal_Bm_ (MHD11)	PIV5 expressing amino acids 22 to 170 of *B. mallei* ATCC 23344 Pal protein	This study
PIV5-BatA	PIV5 expressing amino acids 30 to 307 of *B. mallei* ATCC 23344 BatA protein	[41]
PIV5-Tb	PIV5 expressing Ag85B protein of *M. tuberculosis*	[40]

### Cells and culture conditions for work with PIV5 viruses

BHK21 cells (CCL-10, ATCC) were cultured in DMEM containing Tryptose Phosphate Broth (ThermoFisher Scientific, 10% vol/vol), fetal bovine serum (FBS, 5% vol/vol), penicillin (100 IU/mL), and streptomycin (100 ug/mL). MDBK-A cells (USDA) were grown in DMEM supplemented with 10% FBS and the concentration of penicillin/streptomycin listed above. All cultures were maintained at 37°C with 5% CO_2_.

### Bacterial recombinant DNA methodologies

Standard molecular biology techniques were performed as described elsewhere [36]. Genomic DNA was obtained from *Burkholderia* strains with the Easy-DNA gDNA purification kit (ThermoFisher Scientific). The QIAprep Spin Miniprep kit (Qiagen) was used to isolate plasmid DNA. PCR was carried out with the FailSafe PCR System (epicenter/Lucigen) according to the manufacturer’s recommendations. DNA fragments were cloned into plasmids using T4 DNA ligase and restriction endonucleases purchased from New England Biolabs Inc. The *E. coli* strains EPI300 and EC100D *pir^+^* were used to propagate plasmids.

A DNA fragment of 812 nucleotides (nt) containing the *B. mallei* ATCC 23344 *pal* gene was synthesized with unique 5ʹ-*Eco*RI and 3ʹ-*Nco*I restriction sites for directional cloning (GenScript custom gene synthesis services). The DNA fragment was cloned into the corresponding sites of vector pBHR1 yielding plasmid pPal_Bm_. A larger DNA fragment of 1,332 nt (with additional flanking sequences upstream and downstream of the *pal* gene) was also synthesized and cloned into pACYC184 in a similar manner, producing plasmid pACYCPal_Bm_, and a 0.4 kb zeocin resistance marker from plasmid pEM7/ZEO was inserted into a *Pst*I site near the middle of the *pal* ORF. The resulting construct, pACYPal_Bm_ZEO, was digested with *Dra*I and *Bsu*36I and a 2.2 kb DNA fragment containing the *pal* ORF disrupted with the zeocin cassette was purified using the High Pure PCR product purification kit (Roche Life Sciences), treated with the End-It DNA end repair kit (epicenter/Lucigen), and cloned into the *Eco*RV site of plasmid pKAS46, producing construct pKASPal_Bm_ZEO.

A PCR product encoding amino acids 22 to 170 of the *pal* ORF was amplified from genomic DNA of *B. mallei* ATCC 23344 using the oligonucleotides primers P1 (5ʹ-CTA GCT AGC AAG TCG GGC GTG AAG CT-3ʹ)[*Nhe*I site underlined] and P2 (5ʹ-TTA ATT AAA GTT ACT GTT GAT AGA CGA-3ʹ)[*Pac*I site underlined]. The amplicon was purified, digested with *Nhe*I and *Pac*I, and cloned in the protein expression plasmid pETcoco-1. This approach created the construct pHisPal_Bm_, which contains the *pal* gene product fused to an N-terminal histidine tag (His-tag). A similar strategy was used to clone a PCR product encoding amino acids 47 to 243 of the *B. mallei* ATCC 23344 *lolC* ORF into pETcoco-1, yielding plasmid pHisLolC_Bm_; the primers P3 (5ʹ-CCC AAG CTT GTG CTG TCG GTG ATG AA-3ʹ)[*Hind*III site underlined] and P4(5-CCC TTA ATT AAA GCT CGC GCG CAA CC-3ʹ)[*Pac*I site underlined] were used to generate the amplicon containing the *lolC* gene fragment.

Electroporation was used to introduce plasmids into *E. coli* strains. The plasmids pBHR1∆Dra, pPal_Bm_, and pKASPal_Bm_ZEO were introduced into *B. mallei* via conjugative transfer using the *E. coli* strain S17, as previously outlined [37,38]. The plasmids pHisPal_Bm_ and pHisLolC_Bm_ were sequenced to verify that the cloned DNA did not contain mutations that would result in amino acid substitutions.

### *Construction of* B. mallei *ATCC 23344* pal *mutant strain*

After the transfer of plasmid pKASPal_Bm_ZEO into *B. mallei* ATCC 23344, Polymyxin B resistant colonies were selected for resistance to zeocin and sensitivity to kanamycin. These colonies were then tested by PCR with primers P1 and P2, which produced amplicons of 0.5 kb in WT *B. mallei* ATCC 23344 and 0.9 kb in the *B. mallei pal* KO mutant (data not shown). This 0.4-kb difference in size was consistent with the aforementioned insertion of a zeocin resistance marker into a *Pst*I site near the middle of the *pal* gene and confirmed allelic exchange in the genome of the mutant.

After the transfer of plasmids pBHR1∆Dra and pPal_Bm_ into the *B. mallei pal* KO mutant strain, Polymyxin B resistant colonies were selected for resistance to zeocin and kanamycin. Plasmid DNA was purified from these colonies and tested with restriction endonucleases to authenticate the constructs (data not shown).

### *Construction of PIV5 producing the* B. mallei *Pal protein*

A gene fragment encoding amino acids 22–170 of the *B. mallei* ATCC 23344 Pal protein was synthesized with codon usage optimized for human cells (GenScript custom gene synthesis services). The DNA fragment was cloned between the SH and HN genes of PIV5 in the plasmid ZL185, which contains the entire genome of the virus. The resulting construct, PIV5-Pal_Bm_, was rescued and confirmed by RT-PCR sequencing as described previously [39]. PIV5 viruses expressing *Mycobacterium tuberculosis* protein Ag85B (PIV5-Tb) [40] and the *B. mallei* ATCC 23344 autotransporter BatA (PIV5-BatA) [41] were used as negative and protection benchmark controls in vaccination studies, respectively. WT PIV5, PIV5-Pal_Bm_, PIV5-Tb, and PIV5-BatA viruses were propagated in MDBK cells as reported elsewhere [39].

### Growth curves and plaque assay

Six-well plates seeded with MDBK cells were infected in triplicate with WT PIV5 or PIV5-Pal_Bm_ at a multiplicity of infection (MOI) of 0.01. One hundred μL samples of supernatant were then collected daily over 5 days. The virus titers in supernatant samples were determined by plaque assay as described previously using BHK21 cells [40].

### Animal experiments

Female BALB/c mice (6–8 weeks of age) were obtained from Envigo. Challenge with bacteria was performed via the aerosol route as previously reported [42]. The infected animals were monitored daily for clinical symptoms and humane endpoints were strictly observed. Mice showing signs of intermediate to severe distress were euthanized per AVMA guidelines. At study endpoints, tissues were harvested from euthanized survivors, homogenized, diluted, and spread onto agar plates to determine bacterial burden.

Vaccination with PIV5 viruses was performed intranasally (IN). Mice were first anesthetized intraperitoneally with 250 mg/kg tribromoethanol (Millipore Sigma). The animals were then held in supine position and 5–10 uL droplets of PIV5 virus stock were delivered to the nostrils; a dose of 10^7^ PFU in a final volume of 40 uL was administered. Six weeks later, mice were challenged with 8,000 CFU of *B. mallei* ATCC 23344 (10 LD_50_) via the aerosol route using a Microsprayer device as previously described by our group [42]. The controls in these experiments consisted of age- and weight-matched animals that were vaccinated IN with PBS.

### Antigen preparation and analysis

Whole cell lysates were prepared from plate-grown bacteria suspended in 5 mL PBS to a concentration of 1 × 10^9^ CFU per mL. Bacteria were pelleted by centrifugation, washed once with PBS, resuspended in 0.5 mL Bugbuster HT Protein Extraction Reagent (Millipore Sigma) supplemented with 1 uL/mL of rLysozyme Solution (Millipore Sigma), and incubated for 30 min at 65°C. Western blot analyses were carried out as previously published [19,43,44]. The *E. coli* strain TUNER containing plasmids pHisPal_Bm_ and pHisLolC_Bm_ was used to produce His-tagged Pal_Bm_ and LolC_Bm_ proteins, respectively. Protein expression was induced by the addition of 1 mM isopropyl-β-D-thiogalactopyranoside to broth cultures and incubating for 5 hours. Following this, bacteria were centrifuged and treated with Bugbuster HT Protein Extraction Reagent containing 1 uL/mL of rLysozyme Solution. Both His-tagged Pal_Bm_ and LolC_Bm_ proteins were soluble and purified with a His-Bind Purification kit (Millipore Sigma). Protein concentrations were determined with a BCA Protein assay kit (ThermoFisher Scientific). For ELISA, Immulon 2HB plates (ThermoFisher Scientific) were coated with antigen preparations or paraformaldehyde-inactivated *B. mallei* cells and antibody reactivity was measured as previously outlined [19,43].

To investigate protein expression in cells infected with PIV5 viruses, monolayers of MDBK cells were first infected with PIV5-Pal_Bm_ and WT PIV5 (MOI of 10). After a 24 hr incubation period, cells were lysed with 1X Laemmli Sample Buffer (Bio-Rad) in PBS with 2-mercaptoethanol and heated at 95°C for 5 min. Samples were then resolved on a 4–20% gradient acrylamide gel by SDS-PAGE and transferred to an Amersham Hybond LFP PVDF membrane (GE Healthcare Life Sciences); purified His-tagged Pal_Bm_ protein was included as a control in these experiments. Upon protein transfer, the PVDF membrane was incubated with mouse anti-Pal_Bm_ polyclonal antibodies followed by incubation with Cy3-conjugated goat anti-mouse IgG (Jackson ImmunoResearch). A duplicate PVDF membrane was also probed with a mouse antibody against the PIV5 gene product P/V (Pk) antibody. The reactivity of antibodies with protein bands was visualized using a Typhoon FLA 7000 system (GE Healthcare Life Sciences).

### Antibodies

Immune serum containing antibodies against Pal_Bm_ and LolC_Bm_ was produced by immunizing mice with His-tagged Pal_Bm_ and His-tagged LolC_Bm_ mixed with adjuvant, respectively. Mice were given three vaccine doses subcutaneously at 14 days interval. Each dose consisted of 25 ug purified protein mixed in a ratio of 1:1 with Freund’s Adjuvant (Millipore Sigma; complete for prime, incomplete for boost). Immune serum was collected via the tail bleed procedure, pooled, and used in Western blot analyses. The monoclonal antibodies Pal_Bm_ MAb#1 and Pal_Bm_ MAb#2 were obtained by fusing spleen cells (from a vaccinated mouse) with Sp2/mIL6 cells (ATCC CRL 2016) as previously reported [19,43]. The supernatant from cultures of cells secreting the antibodies were used in Western blot analyses.

### Macrophage killing assays

The mouse cell line J774A.1 (ATCC TIB-67) was cultured and infected with bacteria as previously outlined by our group [19,37,43,45]. The antibiotic streptomycin was used at a concentration of 50 ug/mL to kill extracellular bacteria.

### Serum killing assays

Plate-grown organisms were used in all experiments. Bacterial suspensions were prepared in buffer supplemented with 5 mM MgCl_2_ and 1.5 mM CaCl_2_, mixed with normal human serum at a final concentration of 25% (vol/vol), and the mixtures were incubated at 37ºC. At time 0, 30, 60, and 120 min, duplicate 10 uL aliquots were removed and plated onto agar medium to determine the number of viable bacteria, as previously reported [46].

### Data analysis

The Kaplan-Meier method was used to plot survival data and the Log-rank Mantel-Cox and Gehan-Breslow-Wilcoxon tests were used to perform statistical analyses. The student’s *t* and Mann–Whitney tests were used to analyze ELISA, bacterial burden data, and cell culture assay results. The two-stage linear step-up multiple *t* test procedure of Benjamini, Krieger, and Yekutieli was used to analyze the data from macrophage and serum killing assays. These analyses were performed using the GraphPad Prism software and *P* values < 0.05 are reported as statistically significant. The method of Reed and Muench [47] was used to calculate LD_50_ values of *B. mallei* WT and recombinant strains.

### Research compliance

All work with live *B. mallei* was performed in BSL3/ABSL3 laboratory space with approvals from the University of Georgia’s Institutional Biosafety and Animal Care and Use Committees (IBC and IACUC, respectively), and in compliance with the rules and regulations of the U.S. Federal Select Agent Program.

## Results

### In silico *characterization of the* B. mallei pal *gene*

Comparative sequence analyses identified the ortholog of the *B. pseudomallei* K96243 *pal* gene on chromosome I of the *B. mallei* ATCC 23344 genome (locus tag BMA2082). The open reading frame (ORF) encompasses 513 nucleotides (nt) and encodes a lipoprotein of 170 amino acids (aa) with a molecular mass of 18, 762. Analyses using the SignalP 5.0 and LipoP 1.0 servers detected a leader peptide at the N-terminus of the *B. mallei* Pal protein (Pal_Bm_) with a signal peptidase II cleavage site between residues 20 and 21 (*i.e*. GALAA^20^ | C^21^KSGV), which would provide a free cysteine residue for lipid modification of the mature protein.

Analysis with NCBI CDD indicated that Pal_Bm_ belongs to the OmpA_C-like Superfamily (domain ID cl28145; E value of 4.39e-64), which comprises peptidoglycan-associated lipoproteins of the Tol-Pal system. Consistent with this finding, orthologs of the *tolQ* (BMA2078), *tolR* (BMA2079), *tolA* (BMA2080), and *tolB* (BMA2081) genes were identified upstream of the *pal* ORF in the *B. mallei* ATCC 23344 genome. This particular arrangement of the genes encoding Tol-Pal system components (i.e. *tolQRABpal*) is conserved in most sequenced genomes of Gram-negative bacteria [32].

NCBI BLAST searches identified *pal* orthologs in the genomes of all *B. mallei* (*n* = 51) and *B. pseudomallei* (*n* > 1, 000) strains that are available through the service. The proteins encoded by these *pal* genes were all identical. An ortholog of *pal* was also identified in the genome of the closely related organism *Burkholderia thailandensis*, with the gene product showing 99% identity. The Pal protein of other *Burkholderia* species, including *cenocepacia, cepacia*, and *multivorans*, exhibited 84% identity with Pal_Bm_.

### In vitro *characterization of the* B. mallei pal *gene*

To study the function of the *pal* gene, we constructed a mutant strain of *B. mallei* ATCC 23344. Mutagenesis was achieved via genomic recombination of a DNA fragment containing the *pal* ORF disrupted with a zeocin resistance cassette. For complementation purposes, the plasmids pBHR1ΔDra (vector control) and pPal_Bm_ (specifies constitutive expression of Pal_Bm_) were introduced in the *pal* knockout (KO) mutant.

**Figure 1.**
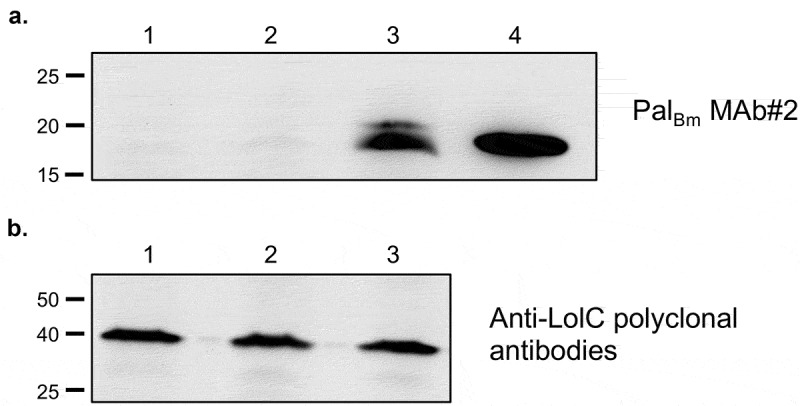
Western blot analysis of *B. mallei* WT and recombinant strains. Whole cell lysates were prepared from WT *B. mallei* ATCC 23344 bacteria (lane 1) and the *pal* KO mutant strain carrying the plasmids pBHR1∆Dra (lane 2) and pPal_Bm_ (lane 3) and analyzed by Western blotting with the monoclonal antibody Pal_Bm_ MAb#2 (panel A) and polyclonal antibodies against the LolC protein (panel B, used as loading control to demonstrate that equivalent amounts of proteins were analyzed). Molecular mass markers are shown to the left in kilodaltons. Lane 4 in panel A corresponds to 5 ug of purified of His-tagged Pal_Bm_ protein (used as positive control to demonstrate reactivity and specificity of Pal_Bm_ MAb#2).

To examine expression of Pal_Bm_, whole cell lysates from wild-type (WT) and recombinant bacteria were analyzed by Western blotting with the monoclonal antibody Pal_Bm_ MAb#2. As anticipated, no reactivity was observed with the mutant carrying the control plasmid pBHR1ΔDra (Figure 1(a), lane 2). We found that the monoclonal antibody does not react with the lysate from WT *B. mallei* ATCC 23344 (Figure 1(a), lane 1); Pal_Bm_-specific polyclonal antisera and a second monoclonal antibody (Pal_Bm_ MAb#1) also failed to show reactivity (data not shown). Western blotting with polyclonal antibodies against the *B. mallei* LolC protein was used to demonstrate that equivalent amounts of lysates were analyzed for all strains (Figure 1(b)). Based on these results, we concluded that Pal_Bm_ is not produced at detectable levels by WT organisms cultured under standard laboratory conditions. As shown in lane 3 of Figure 1(a), introduction of plasmid pPal_Bm_ in the *pal* KO mutant strain results in the production of an 18 kDa protein reacting with antibody Pal_Bm_ MAb#2, which is consistent with the predicted molecular mass of the *B. mallei pal* gene product.

**Figure 2.**
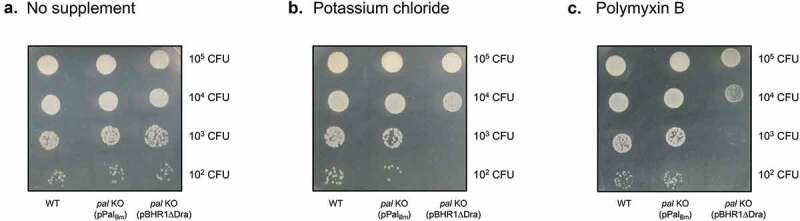
Effect of osmotic and antibiotic stresses on the growth of *B. mallei* WT and recombinant strains. Plate-grown organisms were suspended in PBS to a concentration of 1 × 10^9^ CFU/mL. Ten-fold serial dilutions of bacterial suspensions were then spotted onto agar plates and incubated at 37^º^C for 40 hours. (a) Medium without supplement. (b) Medium supplemented with 100 mM potassium chloride for osmotic stress. (c) Medium supplemented with 20 ug/mL of the antibiotic Polymyxin B. The number of CFU spotted on the agar plates is shown to the right of each panel. Images are representative of 3 independent experiments.

**Figure 3.**
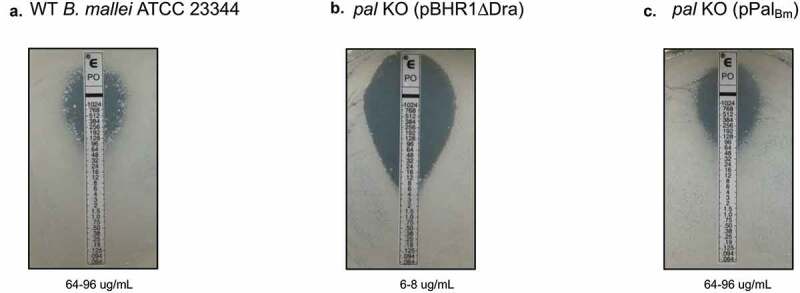
Polymyxin B sensitivities of *B. mallei* WT and recombinant strains. Plate-grown organisms were suspended in PBS to a concentration of 1 × 10^9^ CFU/mL. Portions of bacterial suspensions (10^8^ CFU) were then spread onto agar plates and E strips containing a gradient of Polymyxin B concentrations (0.064–1024 ug/mL, Biomerieux) were applied onto the inoculated medium. After incubation at 37^º^C for 40 hours, minimal inhibitory concentration (MIC) values were determined by reading the intersection of the test strip with the lower part of the ellipse-shaped area of bacterial growth inhibition. (a) WT *B. mallei* ATCC 23344. (b) *pal* KO mutant carrying vector control pBHR1∆Dra. (c) *pal* KO mutant carrying plasmid pPal_Bm_. Images are representative of 3 independent experiments. MIC values are shown at the bottom of each panel.

Mutations in the Tol-Pal system of Gram-negative bacteria typically result in disruption of cell envelope integrity and hypersensitivity to osmotic stress, antimicrobial peptides, detergents, and membrane-acting antibiotics [32]. With this in mind, we assessed the susceptibility of the *B. mallei pal* KO mutant to high concentration of potassium chloride (osmotic stress) and Polymyxin B (antibiotic). The latter consists of a cationic polypeptide with an attached fatty acid tail and initially binds to negatively charged LPS, allowing the hydrophobic tail to interact with bacterial membranes and destabilize them [48]. Suspensions of *B. mallei* WT and recombinant organisms were serially diluted and spotted onto agar plates supplemented with 100 mM potassium chloride (Figure 2(b)) and 20 ug/mL Polymyxin B (Figure 2(c)). Each dilution was also spotted onto medium without additive to verify that the *pal* mutation does not cause a global growth defect (Figure 2(a)). We found that the *pal* KO mutant exhibits increased sensitivity to both osmotic stress and Polymyxin B compared to WT strain ATCC 23344, as evidenced by lack of visible growth when 10^2^ and 10^3^ CFU of bacteria carrying plasmid pBHR1ΔDra were spotted onto agar plates. Consistent with these results, the calculated minimum inhibitory concentration of Polymyxin B for the *pal* KO mutant harboring the control plasmid is approximately 10-fold lower than for WT organisms (Figure 3(a,b), respectively). Introduction of plasmid pPal_Bm_ in the mutant, and concomitant production of the *pal* gene product, restored susceptibility of the *pal* KO strain to osmotic stress (Figure 2(b)) and Polymyxin B (Figures 2(c) and 3(c)) to WT levels.

**Figure 4.**
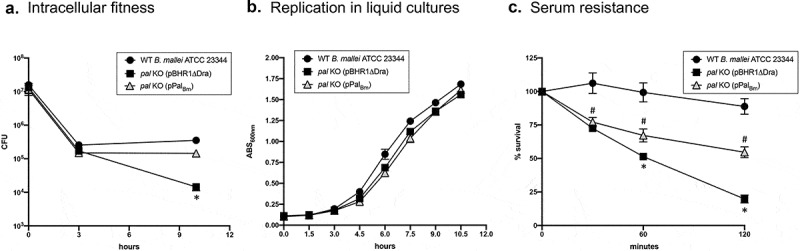
Serum resistance, intracellular fitness, and *in vitro* replication rate in liquid cultures of *B. mallei* WT and recombinant strains. (a) Plate-grown bacteria were suspended in PBS and used to infect 3 wells of duplicate tissue culture plates seeded with murine J774 macrophages (multiplicity of infection of 10:1). The infected cells were incubated for 1 hour at 37^º^C to allow phagocytosis of the bacteria, washed, and treated with antibiotic for 2 hours to kill extracellular bacteria. Cells from one tissue culture plate were lysed, diluted, and plated onto agar medium to determine the number of bacteria phagocytized. The other tissue culture plate was incubated for an additional 7 hours, after which the cells were washed, lysed, diluted, and spread onto agar plates to calculate the number of intracellular organisms. The results are expressed as the mean CFU per well. The assays were performed on 3 separate occasions. The graph shows cumulative results. The error bars correspond to standard errors of the mean. The asterisk indicates that the reduction in the number of intracellular *pal* KO bacteria carrying the vector pBHR1∆Dra at 10 hours post-infection, compared to WT *B. mallei* and the *pal* KO mutant carrying plasmid pPal_Bm_, is statistically significant. The reduction in the number of intracellular *pal* KO bacteria carrying plasmid pPal_Bm_ at 10 hours post-infection, compared to WT *B. mallei*, was not statistically significant. (b) Plate-grown bacteria were suspended in broth to an optical density at wavelength 600 nm (ABS_600 nm_) of 0.1. Following this, suspended bacteria were incubated at 37^º^C with shaking (200-rpm) and the optical density of cultures was measured at the indicated time intervals. Strains were tested on at least 2 separate occasions. The graph shows cumulative results. The error bars correspond to standard errors of the mean. (c) Plate-grown bacteria were suspended in PBS supplemented with Mg^+2^ and Ca^+2^ and aliquots containing 10^3^ CFU were placed in duplicate tubes. Following this, normal human serum was added to the bacteria at a final concentration of 25% and the mixtures were incubated at 37^º^C. Portions were removed at the indicated time points and plated onto agar medium to determine the number of viable organisms. The results are expressed as the mean percentage of the original inoculum (time 0 min) remaining at each time point. The assays were performed on 4 separate occasions. The graph shows cumulative results. The error bars correspond to standard errors of the mean. The asterisk indicates that the reduction in the number of *pal* KO bacteria carrying the vector pBHR1∆Dra and surviving exposure to serum, compared to WT *B. mallei* and the *pal* KO mutant carrying plasmid pPal_Bm_, is statistically significant. The hashtag indicates that the reduction in the number of *pal* KO bacteria carrying plasmid pPal_Bm_ and surviving exposure to serum, compared to WT *B. mallei*, is statistically significant.

The ability to thrive within host cells is an important pathogenicity trait of *B. mallei* [13,14,49,50]. Thus, we determined the intracellular fitness of the *pal* KO mutant using J774 murine macrophage cells. These experiments established that the *pal* mutation substantially reduced fitness over a period of 7 hours (Figure 4(a)). By dividing the number of viable intracellular organisms at the experimental endpoint (CFU at 10 hours) by the number of phagocytized bacteria (CFU at 3 hours), fitness indexes of 1.38 and 0.08 were calculated for the WT strain and the *pal* KO mutant carrying control plasmid pBHR1∆Dra, respectively. To complement these assays, we compared the growth of the strains in broth medium and found that they replicated at similar rates (Figure 4(b)). These data indicate that the intracellular fitness phenotype of the *pal* KO mutant is not the result of a generalized growth defect. Introduction of plasmid pPal_Bm_ in the mutant and constitutive production of the Pal_Bm_ protein restored intracellular fitness to WT levels (Figure 4(a), calculated fitness index of 0.97). Taken together, these data demonstrate a role for Pal_Bm_ in the ability of *B. mallei* to survive and replicate inside host cells.

Serum resistance is another important pathogenicity trait of *B. mallei*, specifically resistance to complement membrane attack complex deposition and cell lysis [51,52]. Given the role of Tol-Pal system components in maintaining the integrity of the bacterial cell envelope and the increased sensitivity of the *B. mallei pal* KO mutant to osmotic stress and the membrane-acting antibiotic Polymyxin B (Figure 2), we compared the susceptibilities of WT and recombinant strains to normal human serum *in vitro*. As shown in Figure 4(c), nearly 90% of WT organisms survived exposure to serum for 120 min. In contrast, only 20% of *pal* KO mutant bacteria carrying the vector control pBHR1∆Dra remained viable. The increased sensitivity of the mutant to complement-mediated lysis was detected as early as 30 min upon exposure to serum (72% survival compared to 106% for WT strain ATCC 23344). Complementation of the *pal* mutation with plasmid pPal_Bm_ partially restored serum resistance. No statistically significant difference was observed between survival of the mutant carrying plasmids pBHR1∆Dra and pPal_Bm_ after 30 min incubation with serum (72–77% survival), but bacteria constitutively producing Pal_Bm_ show increased viability after 60 min (67% survival compared to 50% for the mutant carrying the control plasmid) and at the experimental endpoint (55% survival compared to 20% for control organisms). These data are consistent with the *pal* mutation causing a defect in the integrity of the *B. mallei* cell envelope, which in turn sensitizes the organism to complement-mediated lysis.

### In vivo *characterization of the* B. mallei pal *gene*

**Figure 5.**
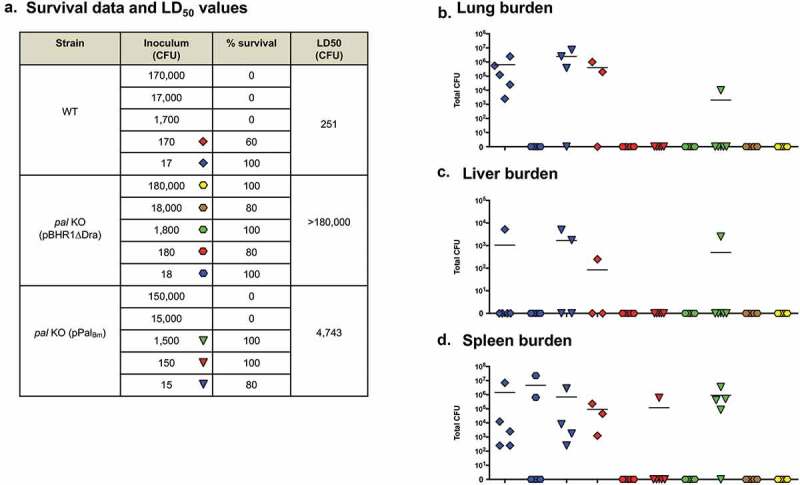
Median lethal dose comparison for *B. mallei* WT and recombinant strains. BALB/c mice were inoculated with a Microsprayer device to aerosolize the indicated numbers of bacterial CFU directly into the lungs (*n* = 5 mice/dose). The animals were then monitored daily for clinical signs of illness and morbidity. (a) Survival data and calculated LD_50_ values. (b–d) Tissues were collected from mice that survived challenge (day 22), homogenized, diluted, and spread on agar plates to determine bacterial loads. The symbols represent data from individual animals; horizontal lines represent the mean total number of CFU for each group.

**Figure 6.**
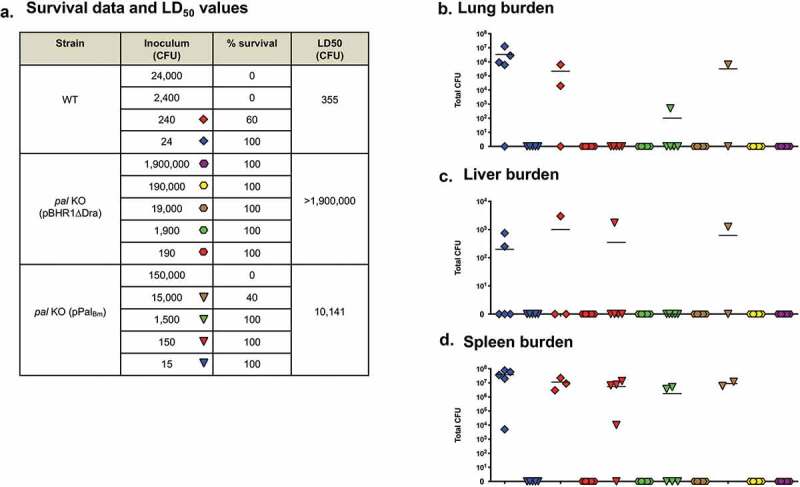
Median lethal dose comparison for *B. mallei* WT and recombinant strains (repeat experiment). BALB/c mice were inoculated with a Microsprayer device to aerosolize the indicated numbers of bacterial CFU directly into the lungs (*n* = 5 mice/dose). The animals were then monitored daily for clinical signs of illness and morbidity. (a) Survival data and calculated LD_50_ values. (b–d) Tissues were collected from mice that survived challenge (day 27), homogenized, diluted, and spread on agar plates to determine bacterial loads. The symbols represent data from individual animals; horizontal lines represent the mean total number of CFU for each group.

Based on the results of *in vitro* intracellular fitness and serum killing assays, we hypothesized that the *pal* gene contributes to pathogenesis *in vivo*. To test this, we calculated the median lethal dose (LD_50_) of the *pal* KO mutant strain in a mouse aerosol infection model. At study endpoints, we collected tissues from survivors and determined bacterial loads as an indicator of *in vivo* fitness. Compared to WT organisms, the calculated LD_50_ value for *pal* KO bacteria carrying the control plasmid pBHR1∆Dra was >5,000-fold higher (Figures 5(a) and 6(a)). No *B. mallei* organisms were cultured from the lungs, spleen or liver of any of the mice inoculated with the mutant, even those challenged with a high dose of 1.9 × 10^6^ CFU (Panels B, C, and D in Figures 5 and 6). In contrast, the lungs and spleen of all mice inoculated with as few as 17 CFU of the parent strain ATCC 23344 were colonized with *B. mallei* (Figure 5(b,d)). Introduction of the plasmid pPal_Bm_ in the mutant and constitutive production of the Pal_Bm_ protein restored virulence and *in vivo* fitness, but not entirely. The LD_50_ of the complemented *pal* KO strain was found to be approximately 30-fold higher that WT *B. mallei*, and target tissues of many (but not all) survivors challenged with low doses of 15 and 150 CFU were colonized (Figures 5 and 6). Taken together, the data from the two independent LD_50_ experiments demonstrate that the *pal* gene product contributes to the virulence of *B. mallei*.

**Figure 7.**
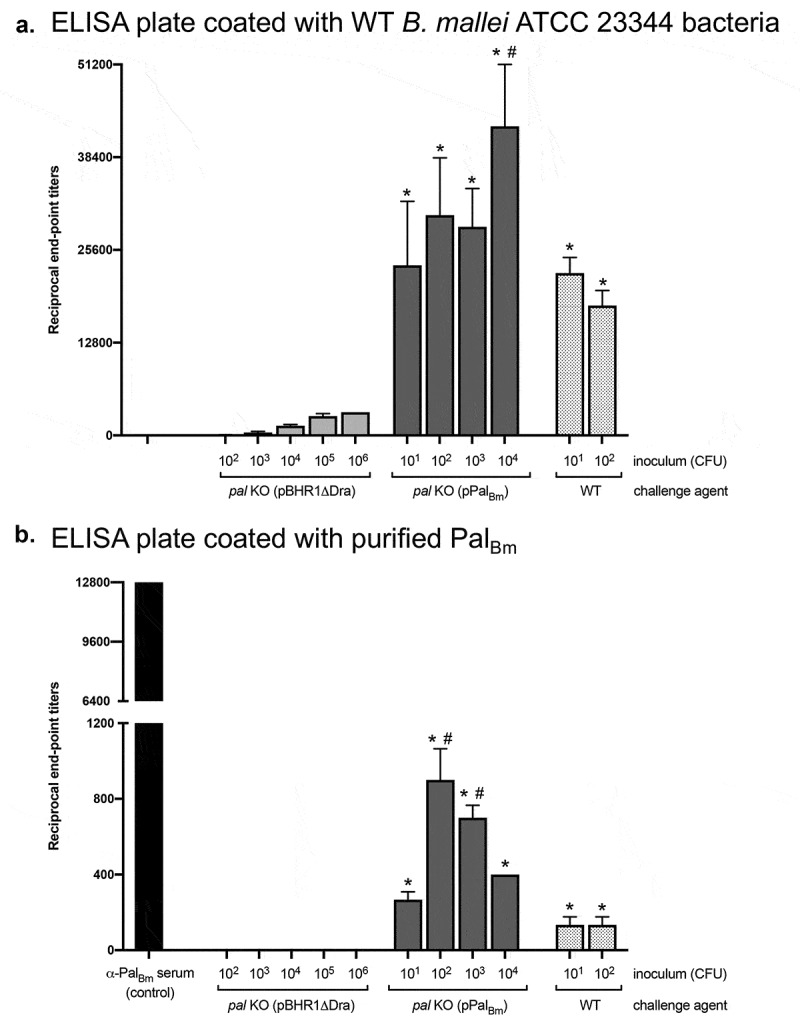
ELISA using serum from mice challenged with *B. mallei* WT and recombinant strains. Serum samples were serially diluted and placed in duplicate wells of plates coated with whole paraformaldehyde-fixed *B. mallei* ATCC 23344 bacteria (panel A) and with purified His-tagged Pal_Bm_ protein (panel B). Alkaline-phosphatase-conjugated goat anti-IgG mouse antibodies (light and heavy chains) were used for detection. Serum samples were tested in duplicate on at least 2 separate occasions. The results are expressed as the mean (plus standard error) reciprocal endpoint titers. Naïve serum was used to establish background reactivity, and the endpoint titers correspond to the highest immune serum dilutions giving ELISA values greater than the mean value of naïve serum plus 3 standard deviations. The labels on the x-axis show the source of immune serum samples (collected from survivors in experiments to determine the LD_50_ of *B. mallei* WT and recombinant strains, see Figures 5 and 6). The asterisks indicate that the increase in serum titers, compared to samples from mice infected with *pal* KO (pBHR1∆Dra) bacteria, is statistically significant. The hashtags indicate that the increase in serum titers, compared to samples from mice infected with WT bacteria, is statistically significant.

To gain insight into the immune response to WT and recombinant strains during infection, we also collected serum from survivors at study end points and measured immunoglobulin titers by ELISA using whole bacteria as well as purified His-tagged Pal_Bm_. As shown in Figure 7(a), challenge with the mutant carrying the vector control pBHR1∆Dra resulted in low titers against whole bacteria (<3,200) compared to titers measured in the serum of mice that survived infection with WT *B. mallei* and *pal* KO organisms harboring plasmid pPal_Bm_. These results are consistent with the considerably reduced virulence and *in vivo* fitness of the mutant, and suggest rapid clearance of bacteria lacking expression of Pal_Bm_. In accordance with Western blot data showing different production levels of the *pal* gene product (Figure 1(a)), serum samples from mice challenged with the complemented mutant were found to contain higher antibody titers against the Pal_Bm_ protein than the serum of survivors infected with WT *B. mallei* (Figure 7(b)). As expected, the serum of mice inoculated with *pal* KO bacteria carrying plasmid pBHR1∆Dra did not show detectable titers of Pal_Bm_-specific antibodies. Collectively, these data show that *B. mallei* produces Pal *in vivo* during infection, which in turn elicits production of Pal_Bm_-specific antibodies. The data also suggest that the extent of the antibody response against Pal_Bm_ correlates with the amount of protein produced by bacteria.

To evaluate the usefulness of the mutant as a live attenuated vaccine, groups of mice were inoculated with the *pal* KO strain and, 4 weeks later, were infected with ~10LD_50_ of *B. mallei* ATCC 23344 organisms. The results of these experiments are outlined in Table 2 and show that vaccinating mice with the *pal* KO mutant strain does not protect against lethal challenge with WT bacteria.

**Table 2.** Vaccine studies to evaluate protection by the *pal* KO mutant strain.

Treatment	# mice challenged	Survival (10 days post challenge)
*pal* KO vaccination (dose = 190 CFU)	4	0/4
*pal* KO vaccination (dose = 1,900 CFU)	5	0/5
*pal* KO vaccination (dose = 19,000 CFU)	5	0/5
*pal* KO vaccination (dose = 190,000 CFU)	5	0/5
*pal* KO vaccination (dose = 1,900,000 CFU)	5	0/5
PBS vaccination	5	0/5

BALB/c mice were inoculated with the indicated dose of the *pal* KO mutant strain using a Microsprayer device. Thirty days later, the animals were challenged with 6,000 CFU of *B. mallei* ATCC 23344 using the Microsprayer. Age- and weight-matched mice vaccinated with PBS using a were used as controls.

### *Vaccination with a virus vector expressing Pal_Bm_ protects against challenge with* B. mallei

**Figure 8.**
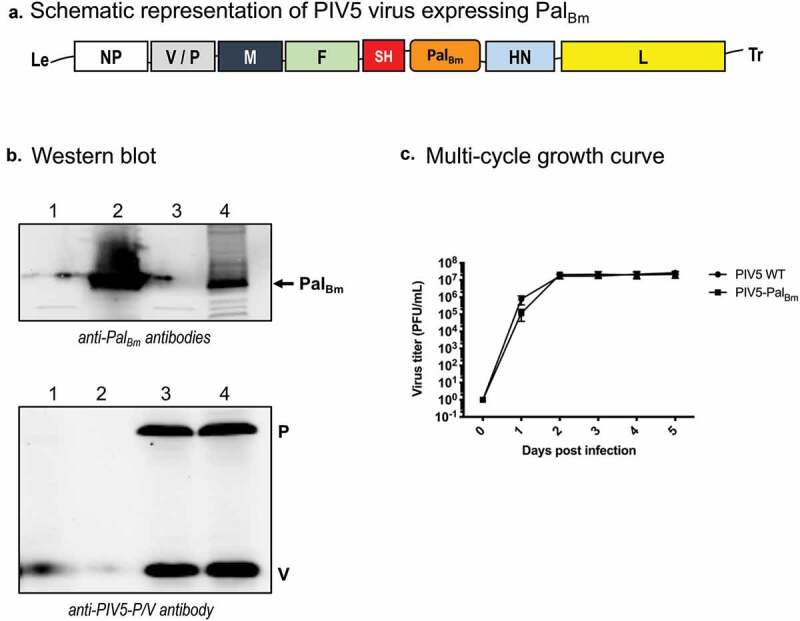
Characterization of recombinant PIV5 virus expressing Pal_Bm_. (a) Schematic of PIV5-Pal_Bm_ vaccine construct. NP, nucleoprotein; V, V protein; P, phosphoprotein; M, matrix protein; F, fusion protein; SH, small hydrophobic protein; HN, hemagglutinin-neuraminidase protein; L, RNA-dependent RNA polymerase; Pal_Bm_, *B. mallei* Pal protein residues 22–170. (b) Equivalent protein amounts of lysates from cells infected with PIV5-Pal_Bm_ and PIV5 viruses were analyzed by Western blotting with mouse Pal_Bm_-specific polyclonal and PIV5-P/V antibodies. Lane 1 = mock infected cells; Lane 2 = purified His-tagged PalBm; Lane 3 = cells infected with WT PIV5 virus; Lane 4 = cells infected with PIV5-Pal_Bm_ virus (c) MDBK cells were infected in triplicate with PIV5-Pal_Bm_ and PIV5 viruses at an MOI of 0.01. Aliquots of supernatant from the infected cell cultures were collected at 24-hour intervals for a period of 5 days post-infection and titers were determined by plaque assays using BHK21 cells. The results are expressed as the mean (± standard error of the mean) log10 PFU/mL. There was no statistically significant difference between viruses.

Based on the aforementioned *in vitro* and *in vivo* data, we hypothesized that delivering Pal_Bm_ with a virus vaccine vector system would elicit excellent protective immunity. This premise is supported by our studies showing that vaccination with Parainfluenza Virus 5 (PIV5) expressing the conserved *Burkholderia* autotransporter protein BatA protected 74% and 60% of mice against death upon infection with *B. mallei* and *B. pseudomallei*, respectively [41]. Therefore, a gene fragment corresponding to amino acids 22–170 of Pal_Bm_ was cloned in PIV5 using a well-established reverse genetics approach developed by our group (Figure 8(a)) [39–41,53]. To verify that the recombinant construct drives expression of the Pal_Bm_ protein, lysates from MDBK cells infected with PIV5-Pal_Bm_ and WT PIV5 viruses were tested by Western blotting. As shown in Figure 8(b), Pal_Bm_-specific antibodies did not react with the control WT PIV5 lysate but reacted with a protein of 18-kDa in cells infected with PIV5-Pal_Bm_. Purified His-tagged Pal_Bm_ protein was used as positive control. An antibody against the PIV5 P/V gene product was also used as loading control to show that comparable amounts of the lysates were analyzed. The replication rates of the viruses were compared in a multi-cycle growth curve experiment over a period of 5 days and found to be equivalent (Figure 8(c)), with PIV5-Pal_Bm_ reaching similar titers as WT PIV5. Hence, Pal_Bm_ expression does not reduce *in vitro* infectivity or fitness of PIV5. The genome of PIV5-Pal_Bm_ was also sequenced to verify that the *pal* gene fragment was properly cloned in its intended location (data not shown).

**Figure 9.**
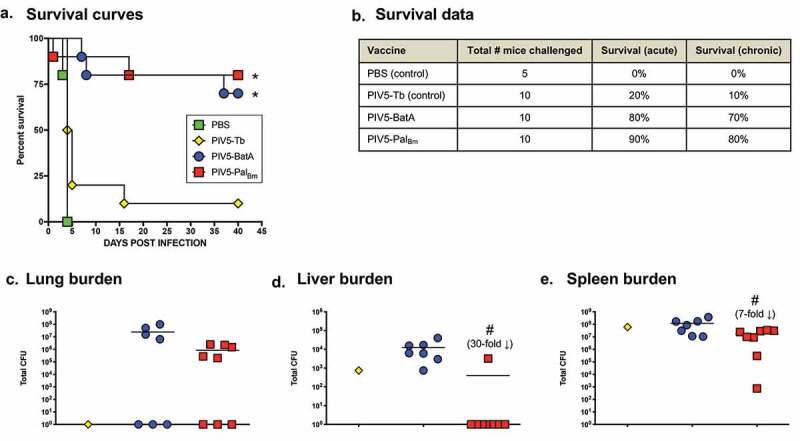
Vaccination with PIV5-Pal_Bm_ provides protection against challenge with a lethal dose of WT *B. mallei*. BALB/c mice vaccinated intranasally with PIV5 viruses were challenged with 10 LD_50_ of *B. mallei* ATCC 23344 using a Microsprayer device and monitored daily for clinical signs of illness and morbidity. (a) Kaplan-Meier survival curves. (b) Survival data during the acute (days 1 through 10 post-challenge) and chronic (days 11 through 40 post-challenge) phases of infection. (c–e) At study end-points, tissues collected from survivors were homogenized, diluted, and spread on agar plates to determine bacterial loads. Symbols represent individual animals; horizontal lines show the mean total CFU for each group. The asterisks indicate that the survival curves were found to be significantly different from mice vaccinated with PBS and the control vaccine PIV5-Tb. The hashtags indicate that the decrease in bacterial burden, compared to mice vaccinated with PIV5-BatA, is statistically significant. Although a 30-fold decrease in lung bacterial burden was observed between mice vaccinated with PIV5-Pal_Bm_ and PIV5-BatA, this decrease was not statistically significant (panel C).

To investigate whether delivering Pal_Bm_ with a virus vector system elicits protection, mice were vaccinated intranasally (IN) with PIV5-Pal_Bm_ and, six weeks later, infected via the aerosol route with *B. mallei* ATCC 23344. Control groups consisted of age- and weight-matched mice treated with PBS, animals immunized with the aforementioned efficacy benchmark PIV5-BatA, and mice vaccinated with a PIV5-Tb construct expressing *Mycobacterium tuberculosis* protein Ag85b (PIV5 vector control) [40]. As shown in Figure 9(a,b), all PBS control mice reached humane endpoints within 5 days post-challenge. Likewise, animals vaccinated with the control virus PIV5-Tb rapidly succumbed to infection and showed negligible survival. In contrast, immunization with PIV5-Pal_Bm_ protected 90% and 80% of mice against death during acute and chronic infection, respectively, which was comparable to the survival rates observed in animals vaccinated with the efficacy benchmark control virus PIV5-BatA. At study endpoints, we determined bacterial loads in target organs of survivors and discovered that no *B. mallei* was present in the liver of *n* = 7 out of 8 mice immunized with PIV5-Pal_Bm_ whereas all animals given the PIV5-Tb and PIV5-BatA vaccines were colonized with *B. mallei* (Figure 9(d)). Three mice from each cohort vaccinated with PIV5-BatA and PIV5-Pal_Bm_ had no bacteria in the lungs and the sole survivor immunized with the control PIV5-Tb vaccine had also cleared the agent (Figure 9(c)). All mice were colonized in the spleen (Figure 9(e)). Collectively, the data show that a single dose of PIV5-Pal_Bm_ provides excellent survival against *B. mallei* lethal aerosol challenge and promotes bacterial clearance from target organs, in particular the liver.

## Discussion

Published work by other groups and the data herein underscore the value of the Pal protein for developing countermeasures against highly pathogenic *Burkholderia* species. The *pal* gene is present in the genomes of all *B. mallei* and *B. pseudomallei* isolates sequenced to date and the encoded proteins are identical. We showed that Pal_Bm_ plays a role in *B. mallei* intracellular fitness and serum resistance (Figure 4), and is expressed *in vivo* as evidenced by the titer of Pal_Bm_-specific antibodies in the serum of mice infected with the organism (Figure 7(b)). Moreover, we showed that a *B. mallei* mutant lacking the protein is greatly attenuated in virulence and completely cleared from host tissues even at high challenge doses (Figures 5 and 6). Immune response against the Pal protein of *B. pseudomallei* (Pal_Bp_) has been linked to resolution of chronic and recurrent melioidosis in human patients [26]. Experimental subunit vaccines containing purified Pal_Bp_ were also shown to provide partial protection against lethal intraperitoneal challenge with *B. pseudomallei* in mice [29,30], and our data indicate that delivering Pal_Bm_ with a viral vaccine system elicits outstanding protection against aerosol exposure to *B. mallei* (Figure 9). Thus, Pal possesses many qualities of a strong candidate for devising medical intervention strategies and targeting the protein may interfere with the ability of *B. mallei* and *B. pseudomallei* to establish themselves in the host, persist, and cause infection.

We discovered that *B. mallei* ATCC 23344 does not produce detectable amounts of Pal when cultured under routine laboratory conditions. These results are consistent with published proteomics data for the organism. Specifically, Schell and colleagues analyzed outer membrane protein preparations from ATCC 23344 cells grown under multiple conditions and their data show that Pal (*i.e*. BMA2082) was present in very low abundance and only in some of the conditions tested [54]. A review of the literature indicated that Pal is produced at readily detectable levels by most organisms in which expression of the protein has been studied including *E. coli* [55], *Haemophilus influenzae* [56], *Erwinia chrysanthemi* [57], *Pseudomonas aeruginosa* [58], and *Haemophilus ducreyi* [59]. Hence, the *pal* gene appears to be more stringently regulated in *B. mallei*.

Despite low expression levels, Pal_Bm_ significantly contributes to maintaining the integrity of the *B. mallei* cell envelope as illustrated by the hypersensitivity of the *pal* KO mutant to osmotic stress and the membrane-acting antibiotic Polymyxin B (Figures 2 and 3). The plasmid pPal_Bm_, which specifies constitutive production of Pal_Bm_, restored susceptibility of the *pal* KO strain to osmotic stress and Polymyxin B to WT levels. The *in vitro* phenotypic characterization of WT and recombinant bacteria also revealed a connection between Pal_Bm_ and the ability of *B. mallei* to thrive intracellularly and withstand complement-mediated killing. While the pPal_Bm_ construct restored intracellular fitness to parental levels, the serum resistance defect was partially complemented (Figure 4). Similar observations were previously made in a *Klebsiella pneumoniae* strain deleted for *pal* [60]. The mutant exhibited reduced protection from serum and neutrophil killing; the serum resistance defect was partially rescued by complementation with an intact copy of the *K. pneumoniae pal* gene and intracellular fitness was fully restored to WT levels. Likewise, a mutation in the gene encoding the *Haemophilus influenzae* Pal ortholog P6 protein was shown to result in increased sensitivity to normal human serum and complementation partly restored resistance [56]. Lack of full complementation of the serum resistance phenotype of the *pal* KO mutant is likely due to a gene dosage effect and overproduction of Pal_Bm_ by the complemented strain (Figure 1(a)). The disproportionate amounts of Pal_Bm_ in the cell envelope may have the unintended effect of destabilizing bacterial membranes and sensitizing the organism to complement-mediated lysis.

Several studies have highlighted a significant role for Pal in the pathogenesis of bacterial infection. For instance, the virulence of a *pal* deletion mutant of *Burkholderia cenocepacia* was reduced by 88-fold in a *Galleria mellonella* larvae model [61]. Deletion of the *pal* gene increased the LD_50_ of *K. pneumoniae* in a mouse model of intraperitoneal infection from less than 1 × 10^2^ CFU for WT organisms to ~2 × 10^5^ CFU [60]. An isogenic *pal* mutant strain of *H. ducreyi* was also found to be considerably attenuated in a human challenge model [59]. Our data demonstrate that a mutation in the *pal* gene of *B. mallei* ATCC 23344 is highly attenuating, as mice inoculated with 2, 500 LD_50_ all survived challenge and completely cleared the organism from target tissues (Figures 5 and 6). Introduction of the plasmid pPal_Bm_ in the *pal* KO mutant restored virulence, but not back to parental levels; the LD_50_ of the complemented strain was still approximately 30-fold higher than WT *B. mallei*. In light of our results showing that serum samples from the mice infected with complemented *pal* KO bacteria contain higher antibody titers against Pal_Bm_ than the serum of survivors infected with WT *B. mallei* (Figure 7(b)), we propose that the ostensively incomplete rescuing of the virulence phenotype results from increased immune clearance via targeting of the Pal_Bm_ protein overexpressed by the complemented mutant. Alternatively, partial complementation may be due to gene dosage effect. Our laboratory as well as other groups have reported similar difficulties studying membrane proteins and complementing *in vivo* virulence defects with plasmid-based systems [19,43,62,63]. Collectively, our data indicate that Pal contributes to the virulence of *B. mallei*, although its specific role is not clearly defined yet. We can not exclude the possibility that the intracellular fitness, serum resistance, and *in vivo* virulence phenotypes of the *pal* KO mutant are indirectly caused by a general defect in cell membrane integrity. Additional studies are ongoing to clarify the specific role of the *pal* gene product in these phenotypic traits.

The Pal proteins of several organisms have been shown to possess excellent vaccinogenic potential [32,64,65]. One of the best studied examples is the aforementioned P6 protein, which is considered a lead vaccine candidate for prevention of non-typeable *H. influenzae* infections in humans (acute otitis media in children, sinusitis, exacerbations in COPD patients, pneumonia). Subunit vaccines containing P6 induce protective immune responses in several animal models including an infant rat model of invasive infection [66,67], a rat pulmonary clearance model [68], and otitis media models in chinchillas and mice [69–71]. An analysis of antibody titers to P6 in children has indicated that immune responses to the protein correlate with protection from *H. influenzae* otitis media [72–74]. Moreover, T-cell responses to P6 in COPD patients are associated with protection from exacerbations caused by *H. influenzae* [75]. Consistent with these studies, the antibody response to the Pal protein of *B. pseudomallei* was shown to be 10-fold higher in plasma from human patients who had only one episode of melioidosis than those with recurrent bouts of disease [26]. Experimental subunit vaccines containing Pal_Bp_ were also shown to provide 50% survival in mice challenged intraperitoneally with *B. pseudomallei* [29,30]. Our data complement and expand upon these findings by demonstrating that one dose of the PIV5 viral vaccine vector expressing Pal_Bm_ affords excellent protection against death upon exposure to *B. mallei* lethal aerosol challenge.

The exact mechanism of protection elicited by the PIV5-Pal_Bm_ vaccine is not clear at the moment. The analyses of serum and bronchoalveolar samples from immunized mice indicate little to no antibody titers against Pal_Bm_ (data not shown). Based on these results and our published work using the PIV5 platform to develop bacterial vaccines for *Mycobacterium tuberculosis* [40] and highly pathogenic *Burkholderia* species [41], T-cell responses are likely the predominant effectors of protection in our model system. Future work should compare and contrast the kinetics, levels, and functionality of humoral as well as cellular responses stimulated by the PIV5-Pal_Bm_ vaccine prior to and during infection, test vaccine efficacy against lethal aerosol challenge with *B. pseudomallei*, and investigate prime-boost vaccination approaches to bolster protective immunity and bacterial clearance from target organs. These studies will identify critical immune components associated with protection and guide efforts for the rational design of PIV5-based vaccines targeting Pal_Bm_ and/or other established immunoprotective antigens such as BatA (see Figure 9) [41], with the goal of achieving sterilizing immunity and complete protection against acute and chronic infections by *B. mallei* and *B. pseudomallei*. Interestingly, the Pal protein of *Burkholderia* species *cepacia, cenocepacia*, and *multivorans* exhibit 84% sequence identity with Pal_Bm_. These bacteria form the *Burkholderia cepacia* complex (Bcc) and are important opportunistic pathogens causing infections in people with cystic fibrosis and for which the availability of effective countermeasures is a critical unmet need [76]. With this in mind, it may be possible to expand the use of the PIV5-Pal_Bm_ vaccine to these organisms.

## References

[1] Khan I, Wieler LH, Melzer F, et al. Glanders in animals: a review on epidemiology, clinical presentation, diagnosis and countermeasures. Transbound Emerg Dis. 2013;60(3):204–221. 22630609 10.1111/j.1865-1682.2012.01342.x

[2] Kettle AN, Wernery U. Glanders and the risk for its introduction through the international movement of horses. Equine Vet J. 2016;48(5):654–658. 27288893 10.1111/evj.12599

[3] Carr-Gregory B, Waag DM. Glanders. In: Dembek ZF, editor. Medical aspects of biological warfare. Borden Institute, Office of the Surgeon General, AMEDD Center and School, Texas, US; 2007. p. 121–146.

[4] Wiersinga WJ, Virk HS, Torres AG, et al. Melioidosis. Nat Rev Dis Primers. 2018;4:17107. 29388572 10.1038/nrdp.2017.107 PMC6456913

[5] Perumal Samy R, Stiles BG, Sethi G, et al. Melioidosis: clinical impact and public health threat in the tropics. PLoS Negl Trop Dis. 2017;11(5):e0004738. 28493905 10.1371/journal.pntd.0004738 PMC5426594

[6] Limmathurotsakul D, Golding N, Dance DA, et al. Predicted global distribution of *Burkholderia pseudomallei* and burden of melioidosis. Nat Microbiol. 2016;1:1. 10.1038/nmicrobiol.2015.8 27571754

[7] Losada L, Ronning CM, DeShazer D, et al. Continuing evolution of *Burkholderia mallei* through genome reduction and large-scale rearrangements. Genome Biol Evol. 2010;2:102–116. 20333227 10.1093/gbe/evq003 PMC2839346

[8] Song H, Hwang J, Yi H, et al. The early stage of bacterial genome-reductive evolution in the host. PLoS Pathog. 2010;6(5):e1000922. 20523904 10.1371/journal.ppat.1000922 PMC2877748

[9] Holden MT, Titball RW, Peacock SJ, et al. Genomic plasticity of the causative agent of melioidosis, *Burkholderia pseudomallei*. Proc Natl Acad Sci U S A. 2004;101(39):14240–14245. 15377794 10.1073/pnas.0403302101 PMC521101

[10] Nierman WC, DeShazer D, Kim HS, et al. Structural flexibility in the *Burkholderia mallei* genome. Proc Natl Acad Sci U S A. 2004;101(39):14246–14251. 15377793 10.1073/pnas.0403306101 PMC521142

[11] Van Zandt KE, Greer MT, Gelhaus HC. Glanders: an overview of infection in humans. Orphanet J Rare Dis. 2013;8:131. 24004906 10.1186/1750-1172-8-131 PMC3766238

[12] Wiersinga WJ, Currie BJ, Peacock SJ. Melioidosis. N Engl J Med. 2012;367(11):1035–1044. 22970946 10.1056/NEJMra1204699

[13] Stone JK, DeShazer D, Brett PJ, et al. Melioidosis: molecular aspects of pathogenesis. Expert Rev Anti Infect Ther. 2014;12(12):1487–1499. 25312349 10.1586/14787210.2014.970634 PMC4409121

[14] Galyov EE, Brett PJ, Deshazer D. Molecular Insights into *Burkholderia pseudomallei* and *Burkholderia mallei* Pathogenesis. Annu Rev Microbiol. 2010;64:495–517. 20528691 10.1146/annurev.micro.112408.134030

[15] David J, Bell RE, Clark GC. Mechanisms of disease: host-pathogen interactions between *Burkholderia* species and lung epithelial cells. Front Cell Infect Microbiol. 2015;5:80. 26636042 10.3389/fcimb.2015.00080 PMC4649042

[16] Hatcher CL, Muruato LA, Torres AG. Recent advances in *Burkholderia* mallei and *B. pseudomallei* research. Curr Trop Med Rep. 2015;2(2):62–69. 25932379 10.1007/s40475-015-0042-2 PMC4410361

[17] Lipsitz R, Garges S, Aurigemma R, et al. Workshop on treatment of and postexposure prophylaxis for *Burkholderia pseudomallei* and B. mallei infection, 2010. Emerg Infect Dis. 2012;18(12):e2-e2. online report. 10.3201/eid1812.120638 PMC3557896 23171644

[18] Rhodes KA, Schweizer HP. Antibiotic resistance in *Burkholderia* species. Drug Resist Updat. 2016;28:82–90. 27620956 10.1016/j.drup.2016.07.003 PMC5022785

[19] Zimmerman SM, Dyke JS, Jelesijevic TP, et al. Antibodies against *in vivo*-expressed antigens are sufficient to protect against lethal aerosol infection with *Burkholderia mallei* and *Burkholderia pseudomallei*. Infect Immun. 2017;85:8. 10.1128/IAI.00102-17 PMC5520430 28507073

[20] Khakhum N, Bharaj P, Myers JN, et al. Evaluation of *Burkholderia mallei* Delta*ton B* Delta*hcp1* (CLH001) as a live attenuated vaccine in murine models of glanders and melioidosis. PLoS Negl Trop Dis. 2019;13(7):e0007578. 31306423 10.1371/journal.pntd.0007578 PMC6658008

[21] Norris MH, Khan MSR, Chirakul S, et al. Outer membrane vesicle vaccines from biosafe surrogates prevent acute lethal glanders in mice. Vaccines (Basel). 2018;6:1. 10.3390/vaccines6010005 PMC5874646 29320408

[22] Baker SM, Davitt CJH, Motyka N, et al. A *Burkholderia pseudomallei* outer membrane vesicle vaccine provides cross protection against inhalational glanders in mice and non-human primates. Vaccines (Basel). 2017;5:4. 10.3390/vaccines5040049 PMC5748615 29232837

[23] Torres AG, Gregory AE, Hatcher CL, et al. Protection of non-human primates against glanders with a gold nanoparticle glycoconjugate vaccine. Vaccine. 2015;33(5):686–692. 25533326 10.1016/j.vaccine.2014.11.057 PMC4304905

[24] Gregory AE, Judy BM, Qazi O, et al. A gold nanoparticle-linked glycoconjugate vaccine against *Burkholderia mallei*. Nanomedicine. 2015;11(2):447–456. 25194998 10.1016/j.nano.2014.08.005 PMC4330121

[25] Felgner PL, Kayala MA, Vigil A, et al. A *Burkholderia pseudomallei* protein microarray reveals serodiagnostic and cross-reactive antigens. Proc Natl Acad Sci U S A. 2009;106(32):13499–13504. 19666533 10.1073/pnas.0812080106 PMC2717108

[26] Suwannasaen D, Mahawantung J, Chaowagul W, et al. Human immune responses to *Burkholderia pseudomallei* characterized by protein microarray analysis. J Infect Dis. 2011;203(7):1002–1011. 21300673 10.1093/infdis/jiq142 PMC3068035

[27] Varga JJ, Vigil A, DeShazer D, et al. Distinct human antibody response to the biological warfare agent *Burkholderia mallei*. Virulence. 2012;3(6):510–514. 23076276 10.4161/viru.22056 PMC3524150

[28] Su YC, Wan KL, Mohamed R, et al. A genome level survey of *Burkholderia pseudomallei* immunome expressed during human infection. Microbes Infect. 2008;10(12–13):1335–1345. 18761419 10.1016/j.micinf.2008.07.034

[29] Hara Y, Mohamed R, Nathan S. Immunogenic *Burkholderia pseudomallei* outer membrane proteins as potential candidate vaccine targets. PLoS One. 2009;4(8):e6496. 19654871 10.1371/journal.pone.0006496 PMC2716516

[30] Champion OL, Gourlay LJ, Scott AE, et al. Immunisation with proteins expressed during chronic murine melioidosis provides enhanced protection against disease. Vaccine. 2016;34(14):1665–1671. 26917010 10.1016/j.vaccine.2016.02.038

[31] Gourlay LJ, Peri C, Ferrer-Navarro M, et al. Exploiting the *Burkholderia pseudomallei* acute phase antigen BPSL2765 for structure-based epitope discovery/design in structural vaccinology. Chem Biol. 2013;20(9):1147–1156. 23993463 10.1016/j.chembiol.2013.07.010

[32] Godlewska R, Wisniewska K, Pietras Z, et al. Peptidoglycan-associated lipoprotein (Pal) of gram-negative bacteria: function, structure, role in pathogenesis and potential application in immunoprophylaxis. FEMS Microbiol Lett. 2009;298(1):1–11. 19519769 10.1111/j.1574-6968.2009.01659.x

[33] Duche D, Similarities HL. Differences between colicin and filamentous phage uptake by bacterial cells. EcoSal Plus. 2019;8:2. 10.1128/ecosalplus.esp-0030-2018 PMC11573288 30681066

[34] Simon R, Priefer U, Puhler A. A broad host range mobilisation system for *in vivo* genetic engineering: transposon mutagenesis in gram-negative bacteria. Bio/Technology. 1983;1:784–791.

[35] Skorupski K, Taylor RK. Positive selection vectors for allelic exchange. Gene. 1996;169(1):47–52. 8635748 10.1016/0378-1119(95)00793-8

[36] Sambrook J, Russell DW. Molecular cloning: a laboratory manual. 3rd ed. Cold Spring Harbor Laboratory Press, New York, USA; 2001.

[37] Balder R, Lipski S, Lazarus JJ, et al. Identification of *Burkholderia mallei* and *Burkholderia pseudomallei* adhesins for human respiratory epithelial cells. BMC Microbiol. 2010;10:250. 20920184 10.1186/1471-2180-10-250 PMC2955633

[38] Burtnick M, Bolton A, Brett P, et al. Identification of the acid phosphatase (*acpA*) gene homologues in pathogenic and non-pathogenic *Burkholderia* spp. facilitates TnphoA mutagenesis. Microbiology. 2001;147(Pt 1):111–120. 11160805 10.1099/00221287-147-1-111

[39] He B, Paterson RG, Ward CD, et al. Recovery of infectious SV5 from cloned DNA and expression of a foreign gene. Virology. 1997;237(2):249–260. 9356337 10.1006/viro.1997.8801

[40] Chen Z, Gupta T, Xu P, et al. Efficacy of parainfluenza virus 5 (PIV5)-based tuberculosis vaccines in mice. Vaccine. 2015;33(51):7217–7224. 26552000 10.1016/j.vaccine.2015.10.124 PMC5785232

[41] Lafontaine ER, Chen Z, Huertas-Diaz MC, et al. The autotransporter protein BatA is a protective antigen against lethal aerosol infection with *Burkholderia mallei* and *Burkholderia pseudomallei*. Vaccine: X. 2019;13:100002. 10.1016/j.jvacx.2018.100002 PMC6668238 33826684

[42] Lafontaine ER, Zimmerman SM, Shaffer TL, et al. Use of a safe, reproducible, and rapid aerosol delivery method to study infection by *Burkholderia pseudomallei* and *Burkholderia mallei* in mice. PLoS One. 2013;8(10):e76804. 24098563 10.1371/journal.pone.0076804 PMC3788738

[43] Zimmerman SM, Michel F, Hogan RJ, et al. The autotransporter BpaB contributes to the virulence of *Burkholderia mallei* in an aerosol model of infection. PLoS One. 2015;10(5):e0126437. 25993100 10.1371/journal.pone.0126437 PMC4438868

[44] Bullard B, Lipski SL, Lafontaine ER. Hag directly mediates the adherence of *Moraxella catarrhalis* to human middle ear cells. Infect Immun. 2005;73(8):5127–5136. 16041029 10.1128/IAI.73.8.5127-5136.2005 PMC1201204

[45] Lafontaine ER, Balder R, Michel F, et al. Characterization of an autotransporter adhesin protein shared by *Burkholderia mallei* and *Burkholderia pseudomallei*. BMC Microbiol. 2014;14:92. 24731253 10.1186/1471-2180-14-92 PMC4021183

[46] Aschenbroich SA, Lafontaine ER, Lopez MC, et al. Transcriptome analysis of human monocytic cells infected with *Burkholderia* species and exploration of pentraxin-3 as part of the innate immune response against the organisms. BMC Med Genomics. 2019;12(1):127. 31492148 10.1186/s12920-019-0575-7 PMC6729079

[47] Reed LJ, Muench H. A simple method for estimating fifty percent end points. Am J Hyg. 1938;27:793–797.

[48] Poirel L, Jayol A, Nordmann P. Polymyxins: antibacterial activity, susceptibility testing, and resistance mechanisms encoded by plasmids or chromosomes. Clin Microbiol Rev. 2017;30(2):557–596. 28275006 10.1128/CMR.00064-16 PMC5355641

[49] Willcocks SJ, Denman CC, Atkins HS, et al. Intracellular replication of the well-armed pathogen *Burkholderia pseudomallei*. Curr Opin Microbiol. 2016;29:94–103. 26803404 10.1016/j.mib.2015.11.007

[50] Aschenbroich SA, Lafontaine ER, Hogan RJ. Melioidosis and glanders modulation of the innate immune system: barriers to current and future vaccine approaches. Expert Rev Vaccines. 2016;(9):1–19. 10.1586/14760584.2016.1170598 27010618

[51] Burtnick MN, Brett PJ, Woods DE. Molecular and physical characterization of *Burkholderia mallei* O antigens. J Bacteriol. 2002;184(3):849–852. 11790757 10.1128/JB.184.3.849-852.2002 PMC139525

[52] Larsen JC, Johnson NH. Pathogenesis of *Burkholderia pseudomallei* and *Burkholderia mallei*. Mil Med. 2009;174(6):647–651. 19585782

[53] Chen Z. Parainfluenza virus 5-vectored vaccines against human and animal infectious diseases. Rev Med Virol. 2018;28:2. 10.1002/rmv.1965 PMC7169218 29316047

[54] Schell MA, Zhao P, Wells L. Outer membrane proteome of *Burkholderia pseudomallei* and *Burkholderia mallei* from diverse growth conditions. J Proteome Res. 2011;10(5):2417–2424. 21391724 10.1021/pr1012398 PMC4917286

[55] Michel LV, Shaw D, Hellman J, et al. Dual orientation of the outer membrane lipoprotein Pal in *Escherichia coli*. Microbiology. 2015;161(6):1251–1259. 25808171 10.1099/mic.0.000084 PMC4635515

[56] Murphy TF, Kirkham C, Lesse AJ. Construction of a mutant and characterization of the role of the vaccine antigen P6 in outer membrane integrity of nontypeable *Haemophilus influenzae*. Infect Immun. 2006;74(9):5169–5176. 16926409 10.1128/IAI.00692-06 PMC1594858

[57] Dubuisson JF, Vianney A, Hugouvieux-Cotte-Pattat N, et al. Tol-Pal proteins are critical cell envelope components of *Erwinia chrysanthemi* affecting cell morphology and virulence. Microbiology. 2005;151(Pt 10):3337–3347. 16207916 10.1099/mic.0.28237-0

[58] Lim A Jr., De Vos D, Brauns M, et al. Molecular and immunological characterization of OprL, the 18 kDa outer-membrane peptidoglycan-associated lipoprotein (PAL) of *Pseudomonas aeruginosa*. Microbiology. 1997;143(Pt 5):1709–1716. 9168620 10.1099/00221287-143-5-1709

[59] Fortney KR, Young RS, Bauer ME, et al. Expression of peptidoglycan-associated lipoprotein is required for virulence in the human model of *Haemophilus ducreyi* infection. Infect Immun. 2000;68(11):6441–6448. 11035757 10.1128/iai.68.11.6441-6448.2000 PMC97731

[60] Hsieh PF, Liu JY, Pan YJ, et al. *Klebsiella pneumoniae* peptidoglycan-associated lipoprotein and murein lipoprotein contribute to serum resistance, antiphagocytosis, and proinflammatory cytokine stimulation. J Infect Dis. 2013;208(10):1580–1589. 23911714 10.1093/infdis/jit384

[61] Dennehy R, Romano M, Ruggiero A, et al. The *Burkholderia cenocepacia* peptidoglycan-associated lipoprotein is involved in epithelial cell attachment and elicitation of inflammation. Cell Microbiol. 2017;19:5. 10.1111/cmi.12691 27886433

[62] Cao L, Lim T, Jun S, et al. Vulnerabilities in *Yersinia pestis caf* operon are unveiled by a salmonella vector. PLoS One. 2012;7(4):e36283. 22558420 10.1371/journal.pone.0036283 PMC3340336

[63] Yang X, Thornburg T, Suo Z, et al. Flagella overexpression attenuates *Salmonella* pathogenesis. PLoS One. 2012;7(10):e46828. 23056473 10.1371/journal.pone.0046828 PMC3463563

[64] Lei L, Yang F, Zou J, et al. DNA vaccine encoding OmpA and Pal from *Acinetobacter baumannii* efficiently protects mice against pulmonary infection. Mol Biol Rep. 2019;46(5):5397–5408. 10.1007/s11033-019-04994-2 31342294

[65] Mobarez AM, Rajabi RA, Salmanian AH, et al. Induction of protective immunity by recombinant peptidoglycan associated lipoprotein (rPAL) protein of *Legionella pneumophila* in a BALB/c mouse model. Microb Pathog. 2019;128:100–105. 30550844 10.1016/j.micpath.2018.12.014

[66] Green BA, Quinn-Dey T, Zlotnick GW. Biologic activities of antibody to a peptidoglycan-associated lipoprotein of *Haemophilus influenzae* against multiple clinical isolates of *H. influenzae* type b. Infect Immun. 1987;55(12):2878–2883. 3316025 10.1128/iai.55.12.2878-2883.1987 PMC260001

[67] Yang YP, Munson RS Jr., Grass S, et al. Effect of lipid modification on the physicochemical, structural, antigenic and immunoprotective properties of *Haemophilus influenzae* outer membrane protein P6. Vaccine. 1997;15(9):976–987. 9261944 10.1016/s0264-410x(96)00296-4

[68] Kyd JM, Dunkley ML, Cripps AW. Enhanced respiratory clearance of nontypeable *Haemophilus influenzae* following mucosal immunization with P6 in a rat model. Infect Immun. 1995;63(8):2931–2940. 7622215 10.1128/iai.63.8.2931-2940.1995 PMC173399

[69] DeMaria TF, Murwin DM, Leake ER. Immunization with outer membrane protein P6 from nontypeable *Haemophilus influenzae* induces bactericidal antibody and affords protection in the chinchilla model of otitis media. Infect Immun. 1996;64(12):5187–5192. 8945564 10.1128/iai.64.12.5187-5192.1996 PMC174506

[70] Green BA, Vazquez ME, Zlotnick GW, et al. Evaluation of mixtures of purified *Haemophilus influenzae* outer membrane proteins in protection against challenge with nontypeable *H. influenzae* in the chinchilla otitis media model. Infect Immun. 1993;61(5):1950–1957. 8478084 10.1128/iai.61.5.1950-1957.1993 PMC280788

[71] Sabirov A, Kodama S, Hirano T, et al. Intranasal immunization enhances clearance of nontypeable *Haemophilus influenzae* and reduces stimulation of tumor necrosis factor alpha production in the murine model of otitis media. Infect Immun. 2001;69(5):2964–2971. 11292713 10.1128/IAI.69.5.2964-2971.2001 PMC98249

[72] Hotomi M, Yamanaka N, Saito T, et al. Antibody responses to the outer membrane protein P6 of non-typeable *Haemophilus influenzae* and pneumococcal capsular polysaccharides in otitis-prone children. Acta Otolaryngol. 1999;119(6):703–707. 10587005 10.1080/00016489950180667

[73] Yamanaka N, Faden H. Antibody response to outer membrane protein of nontypeable *Haemophilus influenzae* in otitis-prone children. J Pediatr. 1993;122(2):212–218. 8429433 10.1016/s0022-3476(06)80115-0

[74] Kodama H, Faden H, Harabuchi Y, et al. Cellular immune response of adenoidal and tonsillar lymphocytes to the P6 outer membrane protein of non-typeable *Haemophilus influenzae* and its relation to otitis media. Acta Otolaryngol. 1999;119(3):377–383. 10380746 10.1080/00016489950181422

[75] Abe Y, Murphy TF, Sethi S, et al. Lymphocyte proliferative response to P6 of *Haemophilus influenzae* is associated with relative protection from exacerbations of chronic obstructive pulmonary disease. Am J Respir Crit Care Med. 2002;165(7):967–971. 11934723 10.1164/ajrccm.165.7.2109009

[76] Pradenas GA, Ross BN, Torres AG. *Burkholderia cepacia* complex vaccines: where do we go from here? Vaccines (Basel). 2016;4(2):10. 10.3390/vaccines4020010 PMC4931627 27092530

